# Passive Trunk Exoskeleton Acceptability and Effects on Self-efficacy in Employees with Low-Back Pain: A Mixed Method Approach

**DOI:** 10.1007/s10926-020-09891-1

**Published:** 2020-05-14

**Authors:** S. J. Baltrusch, H. Houdijk, J. H. van Dieën, J. Th. C. M. de Kruif

**Affiliations:** 1 Department of Research and Development, Rehabilitation Center Heliomare, Wijk aan Zee, The Netherlands; 2 Department of Human Movement Sciences, Faculty of Behavioural and Movement Sciences, Vrije Universiteit Amsterdam, Amsterdam, Amsterdam Movement Sciences, Amsterdam, The Netherlands; 3 Faculty of Science, Methodology and Applied Biostatistics, Vrije Universiteit Amsterdam, Amsterdam, The Netherlands

**Keywords:** Lifting device, Technology acceptance, Self-belief, Implementation strategy

## Abstract

*Purpose* Determinants of successfully introducing passive exoskeletons in the working environment to decrease mechanical loading on the back, are acceptability of the device to management and employees, including self-efficacy of employees when using the device. Therefore, the aim of this study was to assess self-efficacy of employees with low-back pain when using an exoskeleton and the acceptability of such a device to these employees and their managers. *Methods* We used a mixed method approach. We quantitatively assessed the change in self-efficacy of 17 employees with low-back pain when performing daily activity tasks with the exoskeleton, using the modified spinal function sort (M-SFS). Qualitatively, we conducted a focus group with employees and a double interview with two managers to add more insight and understandings into changes in self-efficacy and to discuss challenges of implementing an exoskeleton in the working environment. *Results* Self-efficacy significantly increased by 7% when using the exoskeleton. Employees acknowledged the flexibility of the exoskeleton being advantageous to current static external lifting devices, which confirmed the increase of self-efficacy in both static and dynamic tasks. Individual data showed that the increase in self-efficacy was largest for participants, being greatly restricted by their low-back pain. In the focus group, employees confirmed that they are mostly open to wearing the exoskeleton if they suffer from low-back pain. *Conclusion* If potential challenges, e.g. visibility and potential refusal of wearing an exoskeleton are considered in the implementation strategy, acceptability of and self-efficacy in using the passive trunk exoskeleton would be further improved, potentially contributing to reduced risk of low-back pain.

## Introduction

As one of the most common health reasons for work absence [1], affecting 75–85% of workers at some point in their lifetime [2], low back pain (LBP) continues to be an industrial health problem worldwide [3]. Risk factors contributing to an incidence of LBP are known to be multifactorial [4], including biomechanical, psychosocial and individual factors [5, 6].

Exoskeletons have been introduced in the industrial environment [7] in order to reduce biomechanical work-related risk factors, such as high mechanical load due to frequent lifting and forward bending [5, 8, 9]. Several studies have shown efficacy of different passive exoskeletons to decrease spinal loading during controlled lifting, bending, and static holding tasks [10–15]. One of these devices is the SPEXOR exoskeleton [14], which has shown positive effects on functional performance [16] and metabolic costs during lifting [17]. This exoskeleton was used for the present study.

A challenge when introducing exoskeletons in the actual working environment is the acceptability of the device to users. Consumer’s acceptance of technological innovations is described in the Technology Acceptance Model (TAM) [18]. It comprises variables that explain end-users’ behavioural intentions and the individuals’ acceptance of technology. According to this model, major determinants of accepting new technology are perceived ease-of-use and perceived usefulness, indicating that the model deals with end-users’ beliefs. This implies that potential end-users need to be involved when developing and evaluating new devices to reach high acceptability. Such a user-centred approach improves user satisfaction [19] and increases usability and functionality [20]. To collect end-users’ beliefs on the usability and acceptability of an exoskeleton at work, talking to end-users about their working environment is essential. Psychosocial factors at work, such as work pace, job content and job satisfaction [21], might influence the acceptability of introducing new technology at work. Therefore, knowledge on the effect of exoskeletons on such psychosocial factors is valuable for successful design and implementation of the device.

An important determinant of whether new technology will be acceptable to potential users is its effect on their self-efficacy [18]. According to Bandura [22], seIf-efficacy is defined as a person’s confidence to succeed at a given activity, and it also is an important factor in the development of disability as a result of low-back pain. If people show low levels of self-efficacy, they more likely shy away from tasks they perceive as a personal threat in regard to their musculoskeletal disorder [23]. High levels of self-efficacy have been found to be associated with decreased pain and disability levels in patients suffering from chronic pain [24]. A longitudinal study concluded that self-efficacy is an important variable that mediates the relationship between low back pain and disability over time [25]. This suggests the importance of increasing self-efficacy in low-back pain patients. An exoskeleton for the low back that decreases spinal loading during certain activities might increase self-efficacy in low-back pain patients. As a result, the users’ acceptability of the exoskeleton is likely to improve.

In this study, we aimed to involve potential end-users to investigate factors that are important for acceptability of an exoskeleton in the working environment. We used a mixed method design, in which quantitative research and qualitative research are combined to derive complementary information. Acceptability is challenging to assess quantitatively, as there are so many factors playing a role, and it has rarely been done in exoskeleton research. However, self-efficacy, as one important factor, has been shown to be effectively assessable in a quantitative sense. Therefore, in the quantitative part of the study, we specifically aimed to assess the effect of the SPEXOR exoskeleton on self-efficacy, given the importance of self-efficacy as a mediator between low-back pain and disability and in view of its effect on acceptability. In the qualitative part of the study, we used a focus group and interviews to get more insight into the potential of the device to change behavior when suffering from low-back pain and to assess acceptability of the exoskeleton to potential end-users.

## Methods

### Mixed Method Approach

We applied a mixed method approach; thus, quantitative and qualitative methods were integrated to achieve our research aim. We used a serial exploratory design, by first collecting quantitative data, followed by qualitative data collection [26]. This allows to check for convergence in the findings and leads to final inferences that are based on both results [27]. For participants, we approached the Dutch airline company KLM and the Dutch automotive industry Mitsubishi Turbochargers and Engine Europe, aiming to include workers with a history of recurrent low back pain. We recruited employees working in the luggage handling sector and operators working on the assembly lines, respectively. This research complied with the tenets of the Declaration of Helsinki and was approved by the medical ethical committee of VU medical center (VUmc, Amsterdam, The Netherlands, NL57404.029.16). Informed consent was obtained from each participant.

### Quantitative Method

For the quantitative analysis we assessed work-related self-efficacy by using the Modified Spinal Function Sort (M-SFS), a valid and reliable picture-based questionnaire that can be used in patients with chronic musculoskeletal disorders [28]. It consists of 20 daily activities, that involve loading of the spine. The respondents are asked to estimate their physical capacity in regard to their low-back pain by rating the different activities on a scale from 1 (“able”) through 2, 3 and 4 (“restricted”) to 5 (“impossible”). From this scale a total score is calculated, such that high values indicate high self-efficacy (with a max of 80) and low values indicate low self-efficacy (with a min of 0). A M-SFS score < 56 is hypothesized to be predictive for non-return to work in patients with musculoskeletal disorders [29]. The M-SFS has proven to be of advantage in work-related settings [29] and is often use to assess the self-perceived functional capacity of patients with back pain.

#### Participants

Data for this study were obtained from a subgroup of participants that were included in a larger study on the effects of a passive exoskeleton on functional performance [16]. From that population, employees who had a history of low-back pain were included in this study. The final group had an average pain level of 3 (2–5) on a scale from 0 = no pain to 10 = maximum pain. The age, height and body mass of these participants were mean (SD) 43.4 years (7.3 years), 175 cm (7 cm), and 82 kg (14 kg). The 19 participants selected for this study included 8 luggage handlers, 8 operators and 3 people from non-load handling occupations. Data from two participants had to be discarded from the analysis, since these participants failed to comprehend the questions that were asked and therefore could not finish the questionnaire within the recommended time limit.

#### Procedure

**Fig. 1:**
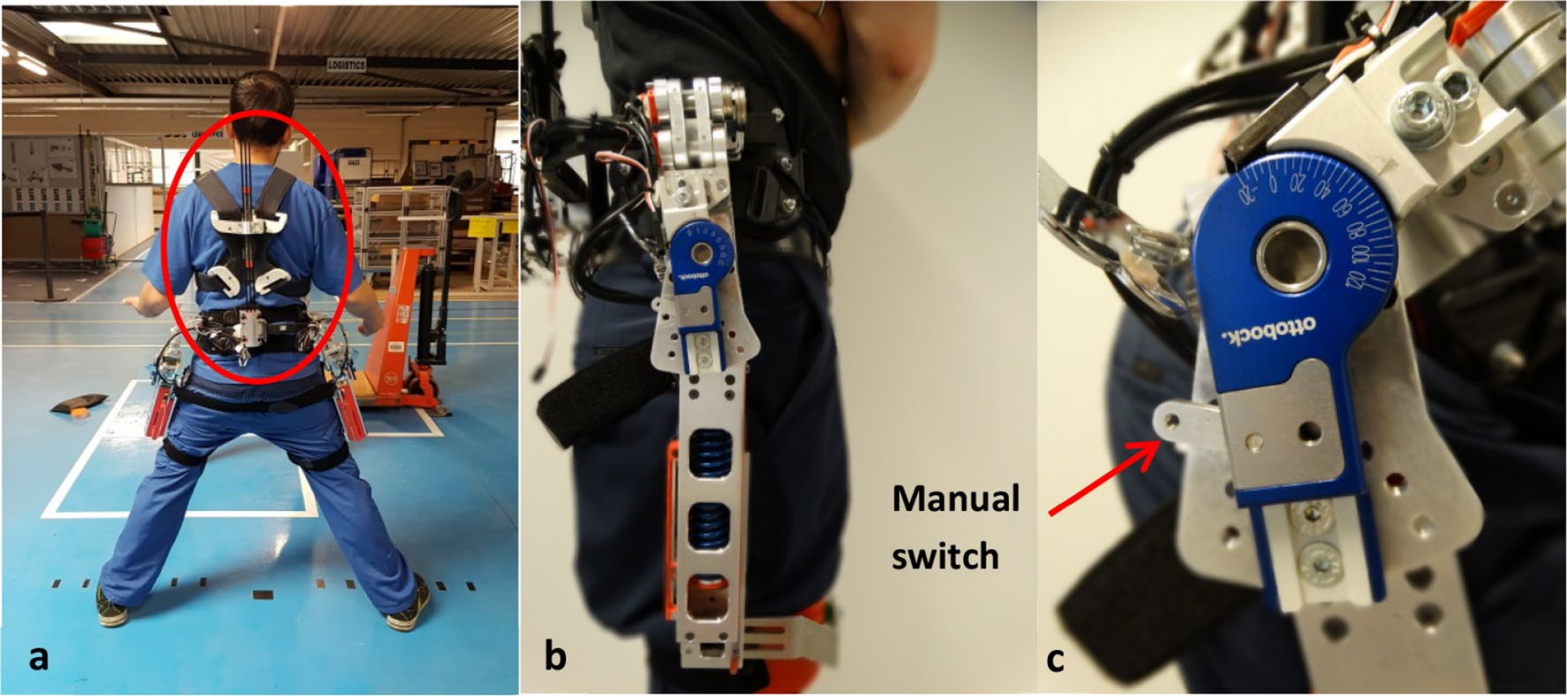
The exoskeleton **a** unloads the back by applying forces at the torso, pelvis, and the thighs, generating a torque by two serially connected passive actuators: an elastic spinal module (**a**, circled red) and a hip actuator (**b**). The implemented clutch allows disengagement of the passive hip actuators, by moving a manual switch **(c)** (Color figure online)

To assess a potential change in self-efficacy with using the exoskeleton, participants were asked to fill in the M-SFS at three different time points: (1) At the beginning of the session (BASE condition), (2) after they received a verbal explanation of the exoskeleton (Fig. 1, more details on the exoskeleton see [14]), thus, they rated their self-efficacy based on their expectations of the exoskeleton (EXPECTATION condition), and (3) after wearing the exoskeleton during a set of daily activities, such as walking, lifting and forward bending with and without the exoskeleton (TRY-OUT condition) (for more information on test battery see [30]). The performed tasks were not the same tasks as depicted in the questionnaire, but provided the participants with sufficient experience of the device to evaluate its potential benefits.

#### Data Analysis

The total score of the M-SFS questionnaire was calculated for the three different assessments and averaged over participants. To find differences between the self-efficacy scores of Baseline, Post-verbal explanation and Post-trying measurement we applied the non-parametric Friedman test. In case of a significant effect of condition, Wilcoxon post-hoc tests were conducted to determine differences between conditions. Alpha of 0.05 was used as the critical level of significance. Statistical analysis was performed using Matlab (R2015b). To get more insight into the effect of the exoskeleton on individual tasks, we categorized the twenty tasks of the M-SFS into 5 categories: (1) lifting, (2) repetitive bending, (3) standing and walking, (4) static forward bending, (5) sitting and (6) others. For each category, we calculated the percentage of participants that scored higher (improved), lower (decreased) and the same (maintained) when wearing the exoskeleton (Post-trying), compared to the control condition (Baseline).

### Qualitative Method

After the quantitative assessment, we conducted a focus group and a double interview to collect perspectives and opinions on the potential use of an exoskeleton in the workplace and the experience with the SPEXOR exoskeleton. Focus groups are used in qualitative research to gather opinions in an interactive group setting. By creating a permissive environment, the researcher encourages participants to share different perceptions without the need to reach consensus [31]. The double interview is a spoken conversation between researcher and two participants, with limited discussion between the participants. The goal of an interview is to capture the diversity of participants’ responses in their own words. Furthermore, it obtains rich and detailed data about individual experiences [32].

#### Participants

The focus group discussion included a sample of the population measured in the quantitative approach. To represent the larger population of luggage handlers, we included employees of different work experience, age, work location and responsibility. Participants were not informed about the results of the quantitative measurements before the focus group study, as this could have influenced their opinion. Since we also aimed to gather opinions on an exoskeleton from the management perspective, a double interview was conducted with one team leader and one process coordinator from KLM, who had been working in the luggage handling sector previously. We did not include them in the focus group, since being from the same working environment, but working in higher positions, their presence could inhibit disclosure among the remaining participants [32].

#### Procedure

Prior to the start of the focus group/interview, participants had to fill in a short questionnaire to obtain demographic details. Subsequently, the focus group discussion and the guided interview were conducted by a moderator (JK) and the main investigator (SB), using a discussion guide (Table 1). To receive information on factors affecting the acceptability of an exoskeleton to employees, the background of these employees needs to be known. We therefore included questions on their working environment, such as working conditions, the use of lifting devices at work and their own strategies to prevent low-back pain. In the double interview, the questions were used to focus on potential implementation strategies and to get answers from the management perspective. The discussion and the double interview were conducted on two different days and lasted between 60 and 90 min. Both sessions were held in Dutch.

**Table 1:** Discussion guide

(1) Round of introductions What are you doing at KLM? How long have you been working at KLM already? Which location are you working at? Can you choose location and type of work?
(2) Working environment Tell us about your working environment Does it vary for different work locations?
(3) How do you use lifting devices? Why do / don’t you use them? How do you deal with low back pain? How do you prevent low back pain?
(4) Try to think back of testing the SPEXOR exoskeleton How did you experience the use of the exoskeleton? What was positive? What was negative? Would you use the exoskeleton if you have low back problems/ as prevention?
(5) What would be the ideal exoskeleton? Wishes/design requirements (conclusion of point 4)

#### Data Analysis

The focus group and the double interview were audio-recorded and discussion notes (memos) were taken during the session. Due to technical problems during the focus group the audio recorder did not record. Therefore, we used the discussion notes to reconstruct the discussion on paper. For trustworthiness and reliability, the discussion notes were sent to the participants of the focus group for verification. Participants confirmed that the discussion notes were a true representation of the topics discussed. The data of the double interview were transcribed verbatim. The participants’ names were replaced by pseudonyms to maintain anonymity. Subsequently, both transcriptions were analysed by the main investigator (SB) using the thematic analysis, a pattern-based analysis that allows to identify and report the salient features of the data [32]. First, categories were developed by repeated close reading of the text. After embedding those into a framework, major themes of the focus group discussion and the interview were identified. The main investigator (SB) performed the coding and analysis. Codes, subthemes and themes were discussed with a team member (JK) until consensus was reached on all categories. Direct quotes used in the discussion were translated into English. Due to data loss we could only use direct quotes from the double interview. We, however, included indirect quotes of the focus group, derived from the reconstructed discussion.

## Results

### Quantitative Results

**Fig. 2:**
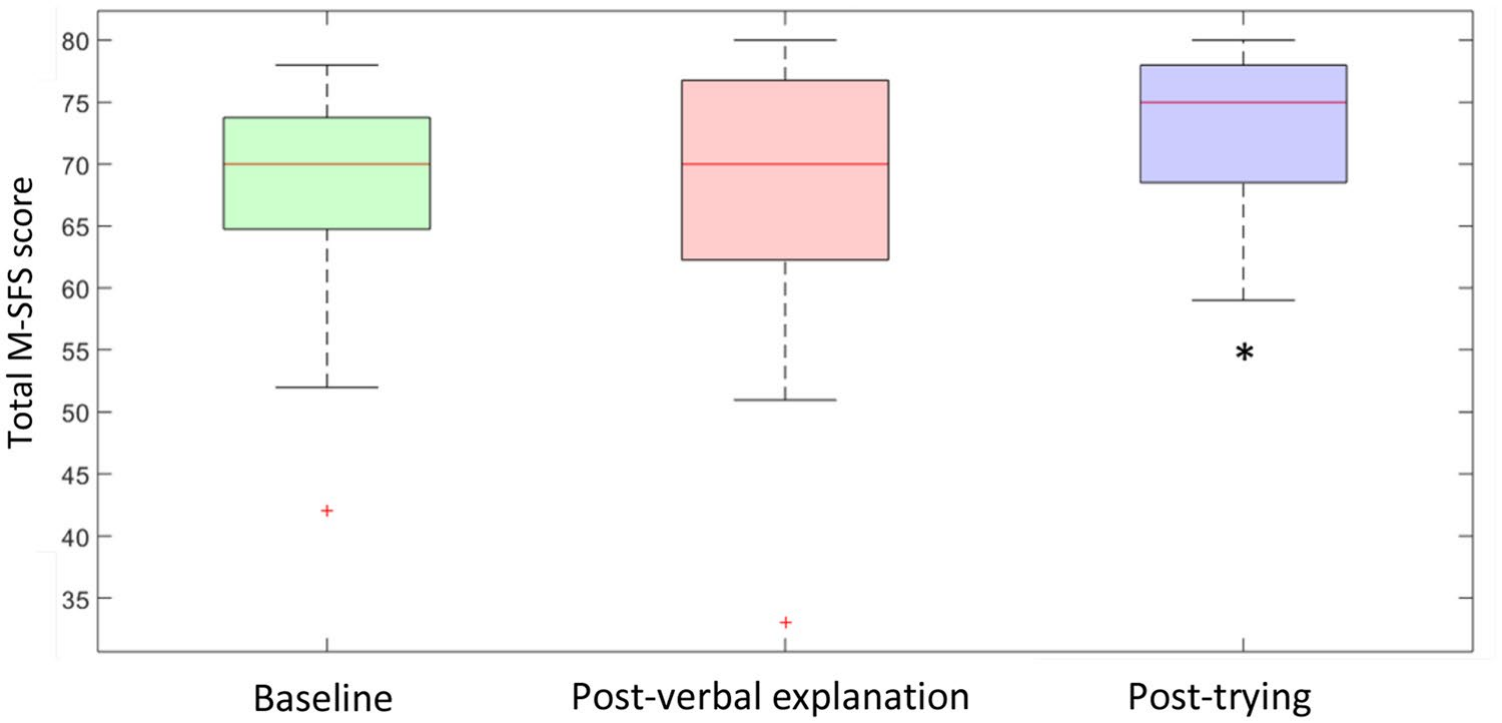
Self-efficacy assessed with the M-SFS questionnaire at the three time points: Baseline, Post-verbal explanation and Post-Trying (The red line represents the sample median. The distances between the tops and bottoms are the interquartile ranges. Whiskers show the min and max values; outliers are represented as a +). *Significant difference in M-SFS score to base measurement (Color figure online)

The assessment of self-efficacy showed a main effect of time point of the measurement (p = 0.02). Post-hoc testing revealed a significant difference between Baseline measurement and Post-trying measurement, with increased total score in the Post-trying measurement (median (IQR): 70 (65–74) vs. 75 (69–78); p = 0.009 (Fig. 2). We did not find a significant difference in self-efficacy between the Baseline measurement and the Post-verbal explanation measurement (70 (65–74) vs. 70 (62–77); p = 0.5.

**Fig. 3:**
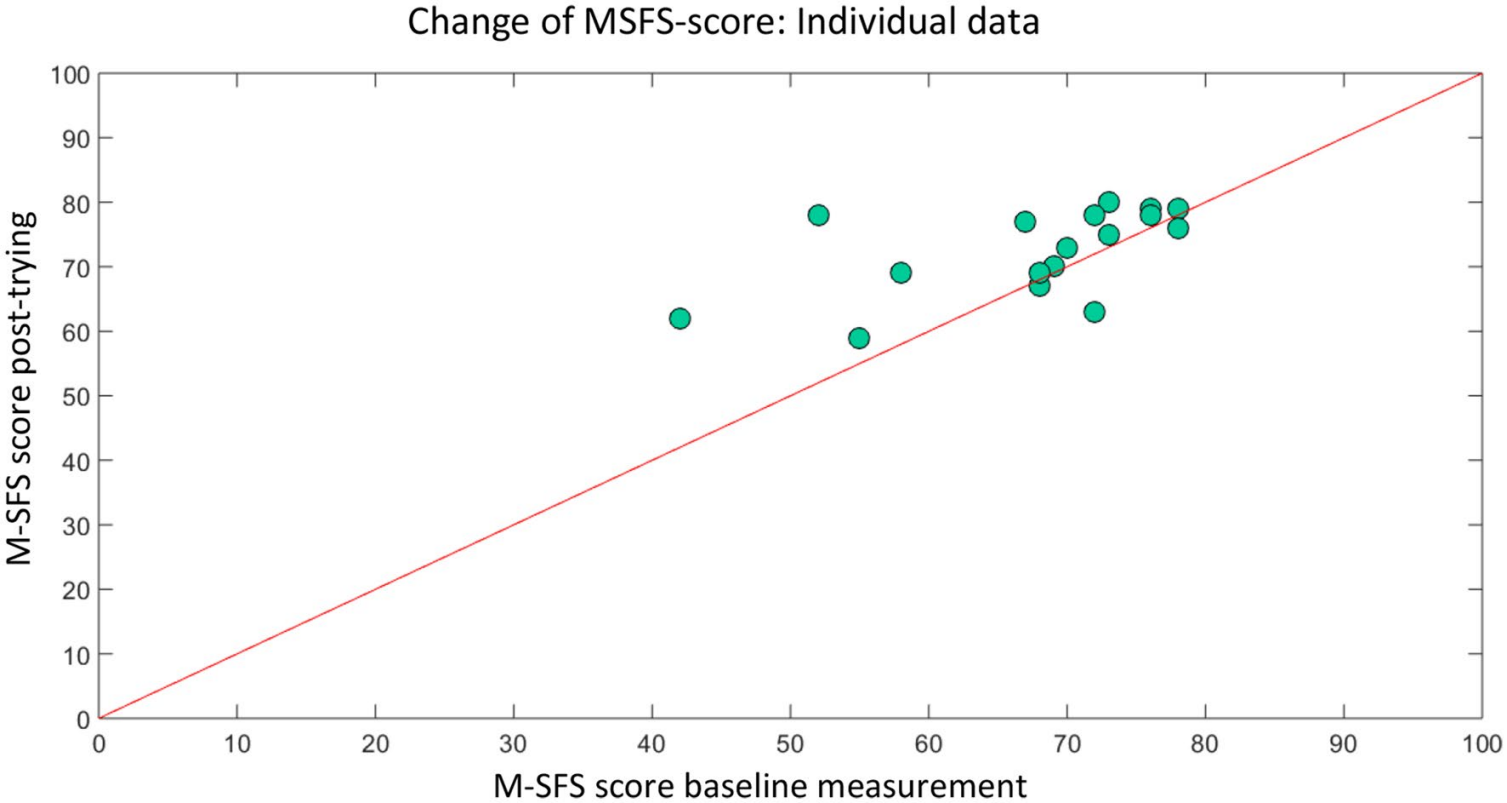
Individual change of M-SFS score from Baseline to post-trying measurement. The data points above the red line represent an increase in total M-SFS score compared to base measurement. The data points below the red line represent a decrease in total M-SFS score compared to base measurement. Low scores in the Basline condition tended to coincide with a large change (Color figure online)

Figure 3 presents the difference of self-efficacy score from Baseline measurement (x-axis) to Post-trying measurement (y-axis) for individual participants. We found that most of the participants started with a MSFS-score higher than the cut-off value (> 56). Specifically, participants with a low MSFS-score at baseline measurement showed the biggest increase in MSFS-score.

**Fig. 4:**
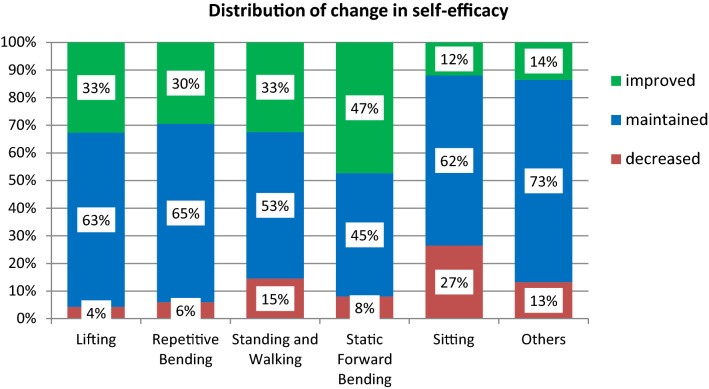
Distribution of change in self-efficacy between BASE condition and TRY-OUT condition for the categorized tasks. Green bars represent an improvement of self-efficacy, blue bars a maintained self-efficacy and red bars a decreased self-efficacy. Percentages of each bar are also presented in the white boxes (Color figure online)

Overall, 28% participants rated higher self-efficacy scores, 12% rated lower self-efficacy scores and 60% of the participants reported small or no change in self-efficacy. The effects of the exoskeleton on self-efficacy in the 5 categories are presented in Fig. 4. For the categories Lifting, Repetitive Bending and Standing and Walking, a third of the participants rated higher self-efficacy after the try-out session. 47% of the participants showed higher scores in the Post-trying session for the task Static Forward Bending. In the categories Sitting and Others, only 12% and 14% of the participants rated higher self-efficacy.

### Qualitative Results

The participant characteristics of the focus group and the double interview are presented in Table 2. Luggage handlers are working at different work locations and are therefore responsible for different tasks. Managers had been working as luggage handlers in the past and sometimes still helped out with loading and unloading luggage.

**Table 2:** Participants’ characteristics

Pseudonym	Group	Age	SE base score	Occupation	Years of employment	Main tasks
Rico	FG	37	76	Luggage handler	9 years	Loading and unloading conveyor belts
Roel	FG	52	68	Luggage handler	26 years	Loading and unloading containers with a robot
Timo	FG	49	70	Luggage handler	21 years	Loading and unloading conveyor belts and containers Driving vehicle
Pim	FG	50	78	Luggage handler	23 years	Loading and unloading conveyor belts and containers Driving vehicle
				Ergo coach		Advising employees and managers
Hendrik	DI	50	72	Manager	21 years	Team leader
Martijn	DI	51	n.a.*	Manager	28 years	Process coordinator

*FG* focus group, *DI* double interview, *SE* self-efficacy

*Not tested

The main categories derived from the focus group discussion and the double interview contributing to the acceptability of an exoskeleton were: “Working conditions”, “Prevention of Low Back Pain”, “Use of Lifting Devices”, “Acceptability and Usability of a spinal exoskeleton” and “Implementation at the Workplace”.

#### Working Conditions

##### High Workload

Luggage handlers agreed on liking their occupation, but considered their workload as high when talking about their working conditions. Due to short times between flights, there is only little time to load or unload luggage, creating high work intensity and short rest time. Additionally, the number of employees had been reduced in recent years, which had caused an increased workload.

The process coordinator confirmed that:

[…] you do have a continuous work pressure. (Martijn, Process Coordinator)

##### Feeling Responsible

Luggage handlers also focused their discussion on feeling responsible for the luggage. They stated that:

Even when luggage was checked in too late and does not have to be considered for loading anymore, they are still trying their best to get it on board.

This also increases workload.

[…] this behaviour is indeed characteristic for the men in the luggage department […] (Martijn, Process Coordinator).

##### Freedom in Task Selection

Luggage handlers acknowledged that:

they have the freedom of choosing their main tasks based on their wishes or current physical problems.

Main tasks are loading, unloading and driving the vehicle to transport luggage to the airplane. When employees report physical problems, the team leader considers that in the distribution of tasks.

#### Use of Lifting Devices

##### Limited Usage

Luggage handlers mainly reported not using the external (off-body) lifting devices that are currently available in the company (see Appendix), because:

If the workload is too high, manual work is faster.

In addition, they reached consensus that using the lifting devices does not make work safer. One luggage handler explained that:

in the past they used a lifting device that works with vacuum to suck a suitcase up. Since it led to various accidents during the work process, we are not allowed to use it anymore.

The Ergo coach remarked

he would not recommend using any of the available lifting devices to newcomers (Pim, Ergo Coach).

In contrast, the team leader defined 3 types of people regarding the use of lifting devices:

[…] I know people who use them I dare to say almost 100 percent, […] you have those who only use them if needed, including me. I see it’s heavy, then I do it with the lifting device. And a few people just want to work without a device. (Hendrik, Team Leader)

##### Limitations

Luggage handlers centred their discussion on different limitations of available external lifting devices. One main argument was:

that lifting with the device is too slow to keep up with the workload. (Timo, Luggage Handler)

The team leader shared that opinion and explained that the use of the device

“[…] depends on how many suitcases are coming into the system. […] and also on the time […]if there are 50 suitcases coming at once […] using the lifting device is good, but then it’s slow […] taking too long […]”. (Hendrik, Team Leader)

Another limitation is:

the rigidity of the device. It does not take into account differences in body length and handedness. This allows for only one way of using it and forces the user to work in a certain way.

Due to the above, the luggage handlers perceived current external devices as a hindrance, rather than as a support. In addition, two of them complained about shoulder problems due to the device’s inflexible behaviour.

One luggage handler shared his experience with working with robots that load big containers automatically. He had to control the robot and in case of mistakes, sorting the suitcases by hand:

He acknowledged that the robot reduced loading on his back, but also remarked the problem of not having a continuous work rhythm. While standing at the control board his muscles get cold. When intervening in the process to sort suitcases he starts lifting with cold muscles. (Roel, Luggage Handler)

The process coordinator was aware of the limitations discussed in the focus group. With regard to the device being too slow, he talked about a

perceived speed of work […], in which the sense of speed is reduced when using such a lifting device. (Martijn, Process Coordinator)

He reported that measurements have been done to compare lifting speed with and without using the lifting device. Results showed that lifting with the device is not slower than without, provided that there is

[…] an acceptable distribution of luggage over time […] (Martijn, Process Coordinator)

He also addressed the inflexible and static behaviour of the lifting device:

[…] such a lifting device is actually a really static thing, it’s hanging there and it can be moved to that side or the other side and that’s pretty much it. (Martijn, Process Coordinator)

Whereas the work of the luggage handler is rather dynamic*:*

And then you, as a human, have to adapt to the device and have to find your way. (Martijn, Process Coordinator)

#### Prevention of Low Back Pain

##### Use of Own Techniques

When focusing the conversation on dealing with low back pain at work, luggage handlers talked about the use of certain lifting techniques to deal with problems they have, but also to prevent an onset of low back pain. One luggage handler explained that:

He is taking an extra step when turning and lifting at the same time, and aiming for an equal distribution of the weight to not overload one side of his body. (Rico, Luggage Handler)

##### Less Physical Workload

Both managers emphasized that they have less problems with their back since they stopped lifting luggage frequently.

[…] less physical work […] that does help to have less back problems. (Martijn, Process Coordinator)

##### Ergo Coaches

Another prevention method that came up in the focus group discussion, is the education of Ergo coaches. Next to their normal work, ergo coaches get trained in the prevention and reduction of physical loading. One of the focus group attendants also works as an Ergo coach. He emphasized in the discussion that:

he gives advice on lifting techniques and assistive devices, but the employees have to decide themselves whether they follow this advice or not. (Pim, Ergo Coach)

The team leader, who also has an advising role, explained:

[…] in the beginning we often said you have to use the lifting device, but lately, nobody does […] it didn’t help. (Hendrik, Team leader)

#### Acceptability and Usability of a spinal exoskeleton

##### Experience During Testing

Luggage handlers shared different experience of the exoskeleton they tested in a previous study. Two of them perceived the lifting task as easier to perform when wearing the exoskeleton, while the other two did not feel a difference. They all agreed on the fact that the exoskeleton is still too heavy, but liked the possibility of switching on and off the support when needed.

##### Time Factor

Luggage handlers were concerned about using the exoskeleton at their workplace when they have to drive the vehicle. Due to the design of the exoskeleton, sitting in the vehicle is not possible. The only solution would be to take it off, but that is time consuming for people who switch between loading, unloading and driving. Taking it on and off has to be fast to make sure no time gets lost.

The process coordinator talked about a previous passive exoskeleton they have tested at workplace and how much time was spent in adapting the exoskeleton to the user. He suggested not using straps to adapt the size of the device, but fixed intervals:

[…] if I know that for my hips for example I need to set it on 3 and for my back on 4 and then I know it’s good for me […] that helps, that really makes a difference in time spent. (Martijn, Process Coordinator)

##### Freedom of Movement

Consensus in the focus group was reached on the fact that the exoskeleton does not interfere with the working task, since it is worn around the body. Luggage handlers acknowledged that:

even when wearing the exoskeleton for support, they can perform their work in their individual manner.

The process coordinator also emphasized the benefit of wearing an on-body device, instead of an off-body lifting device:

[…] if you indeed have something around your body and you can just do your work without restrictions, but it does help in the physical support […] then you perceive it as if you can still do your human movements, in the dynamics like you want it. In terms of speed, of whatever, of how you turn. (Martijn, Process Coordinator)

##### Wear-Resistant and Comfortable Design

The team leader noted that the exoskeleton should be wear-resistant,

[…] you are working [in the luggage hall] you can hit a cart, and then it’s damaged and then it’s not usable anymore. (Hendrik, Team Leader)

and comfortable, considering that it will be worn during a whole working day.

The process coordinator agreed on those points. He suggested to cover the parts of the exoskeleton with a smooth material:

[…] so a practical closed-off structure, so that nothing can get stuck in it. (Martijn, Process Coordinator)

This would also help to deal with limited space, such as handling luggage inside the airplane.

#### Implementation at the Workplace

##### Visibility

The luggage handlers reached consensus that:

they would not mind to be seen when wearing the exoskeleton at the workplace.

The team leader questioned that in the double interview. He remarked that he would see the exoskeleton as part of the work clothes, while his colleagues might have difficulties with being seen.

The process coordinator talked about a

[…] a man’s world […] (Martijn, Process Coordinator)

in which the design of the exoskeleton effects the acceptance to use it. Adapting the colour of the exoskeleton to the colour of the working clothes makes it less visible and more likely to be worn.

##### Contractual Obligation

Both, the focus group and the participants in the double interview, focused their discussion on whether the use of an exoskeleton should be contractually obliged*.* The majority believed that wearing an exoskeleton, especially when suffering from low back pain, is important. Still, luggage handlers agreed on the fact that:

a contractual obligation would be too stringent.

The process coordinator, however, recommended an obligation:

[…] and then not only from the management perspective, but also from the human perspective to protect people against themselves […] (Martijn, Process Coordinator)

He took the example of safety shoes to state his point that by giving employees the choice to wear a “protective” device, the majority would choose against it:

[…] I think if you went into the luggage hall tomorrow and said something like guys if you don’t want to wear safety shoes then you don’t have to, then I think 80% of the people would walk around in sneakers or shoes they like the most. (Martijn, Process Coordinator)

Both, the process coordinator and the team leader thought that one reason for this behaviour is the fact that employees want to be individual and like to wear clothes that express their personality.

##### Prevention or Treatment

An issue that came up in the focus group discussion was whether the exoskeleton would be worn for dealing with current low back problems or to prevent from an onset of low back pain. Luggage handlers were all open to wearing it, but did not see the reason for wearing it preventative. The ergo coach however saw a benefit in a prevention strategy and remarked:

I would advise it [the exoskeleton] (Pim, Ergo Coach)

The process coordinator explained:

I think that it really depends on your personality. I think If I look back at myself 30 years ago, I also wouldn’t have worn it. Now, because you have or had [low-back] complaints, I think, I don’t want these complaints anymore, so in hindsight I would wear it. (Martijn, Process Coordinator)

## Discussion

Using a mixed method approach, we found increased self-efficacy by 7% after trying out the exoskeleton and collected factors from the employee’s and management perspective that might influence the acceptability of an exoskeleton in the working environment. The results indicate the potential acceptability of such devices but also highlight the importance of an adequate implementation strategy and how certain design characteristics, such as comfort, time of adjustment and visibility can influence acceptability of an exoskeleton.

Self-efficacy base scores were relatively high, considering that a self-efficacy score < 56 is hypothesized to be predictive for non-return to work [29]. However, this was expected, since we recruited participants with low-back pain who still performed in their occupation. The unchanged self-efficacy score in the expectation condition indicates that participants at first sight did not expect the exoskeleton to help them in the tasks that were depicted in the questionnaire. The large range of scores over participants in that condition implies that expectations regarding the device were quite different. After trying out the exoskeleton, however, the self-efficacy score significantly increased, indicating that participants in general believed that wearing the device would help them performing the depicted tasks in the questionnaire. The focus group study confirmed this outcome. Luggage handlers were open to try an exoskeleton during their work and saw the potential of an exoskeleton to reduce low-back load. Also, the tasks that were rated with higher self-efficacy when wearing the exoskeleton were similar to the main working tasks described by the employees. The task with the greatest decrease in self-efficacy (Sitting), was also mentioned in the focus group as a potential problem.

Furthermore, we observed increased self-efficacy in not only static, but also dynamic tasks. This can be explained by the qualitative data that describes the flexibility of the exoskeleton as an advantage to current static and inflexible external lifting devices. As an exoskeleton is worn around the body and therefore does not interfere with the workflow, luggage handlers and managers believed in a higher usability and ease of use compared to the current external/off-body systems. These external devices come with several limitations that limit their acceptability. Given the high workload of the employees and their feeling of responsibility, main issues that were mentioned by luggage handlers and managers were perceived slower task performance, and the static, inflexible nature of external devices, conflicting with the dynamic work of a luggage handler.

Although being significant, it can be questioned whether an average change of 5 points in M-SFS score and an increase in self-efficacy by 7% can influence people’s behaviour and increase their confidence in performing work related tasks. According to Trippolini et al. [29], the systematic measurement error for assessing self-efficacy in low-back pain people is around 12–16 points (17–22%). This, however, is only the case for participants having a low self-efficacy (< 60). Participants in the present study had considerably higher scores. Participants who had lowest base scores (40–60) showed the highest increase in self-efficacy. Thus, the findings suggest, that wearing a passive trunk exoskeleton potentially increases self-efficacy in people with low-back pain, having a bigger effect if people show greater restrictions due to their low-back pain. This was confirmed in the focus group, as employees state they are mostly open to wearing the exoskeleton if they suffer from low-back pain, hence when being restricted by their low back. An increase in self-efficacy implies increased confidence to succeed in a given task, implicating decreased low-back pain and disability [24]. This likely leads to higher participation level. Also, increased self-efficacy is expected to increase acceptability of the exoskeleton. When users perceive high self-efficacy regarding assistive technology, they will have a high adoption intention [33].

Quantitative data demonstrated that the majority of the participants showed a high self-efficacy base score, with limited increase in self-efficacy after trying out the exoskeleton. This indicates that they do not feel highly restricted by their low-back pain and thus do not see the need to wear an exoskeleton. Luggage handlers confirmed that outcome, as they did not consider a preventive use of an exoskeleton as needed, but would only wear the exoskeleton when suffering from low-back pain. In contrast, managers stressed the importance of using an exoskeleton as prevention, to protect employees from low-back pain in the long-term. To deal with potential refusal of using the exoskeleton as prevention, they suggested contractual obligation, which might facilitate the managers’ control over using the exoskeleton at work. On the other hand, with an increased self-efficacy when wearing the exoskeleton, as found in the quantitative assessment, acceptability is likely to increase and contractual obligation might be unnecessary. Certainly, these findings demonstrate that the implementation strategy is of importance to convince and support employees to use a spinal exoskeleton at work. The importance of an adequate implementation strategy was also highlighted in a previous study [34], in which design requirements for an exoskeleton used in rehabilitation were discussed with health care professionals and low-back pain patients. Supervision and behavioural coaching were considered as important strategies to ensure the correct use of the exoskeleton. Given the different field of application in the present study, implementation strategies should be more focused on increasing acceptability of the device to guarantee that employees actually use the exoskeleton.

To improve acceptability and usability of an exoskeleton, design requirements from potential end-users should be considered to meet their specific needs [35]. Participants of the focus group and the interview mentioned some design specifications. Given the time constraints and their shift work, the exoskeleton should be easily and quickly adaptable to different body sizes. This might also decrease time pressure and stress, which is a psychological risk factor for an onset of LBP [36]. Additionally, a comfortable and wear-resistant exoskeleton increases its versatility to be implemented at different work locations and for different working tasks. This is in line with previous research that has shown that versatility and comfort are important for user acceptance [9, 30].

Participants discussed potential factors that can negatively affect the acceptability and usability of an exoskeleton in the working environment. Main concerns from the managers were that employees might not use the exoskeleton, as they might not want to be seen with it in a working environment that is dominated by male personalities. If the design of the exoskeleton does not match their personal taste, employees will most likely not accept it. Previous research has described visibility of assistive devices as a disclosure of disability status [37]. The authors suggest to make assistive devices *socially invisible* by choosing a design that matches the social environment. The managers indeed discussed to design the exoskeleton in the colour of the work clothes to make it more *invisible.* Luggage handlers reported they would not mind to be seen with the exoskeleton. Although not entirely consistent, these findings suggest that visibility should be considered when introducing an exoskeleton in the working environment in order to improve acceptability.

Even though the data cannot automatically be generalized to other working environments, we demonstrated potential benefits and challenges from the employees’ and the management perspective when introducing an exoskeleton in a working environment. Self-efficacy base scores were relatively high. Further research is needed to assess whether participants who show greater restrictions due to their low-back pain indeed show greater increase in self-efficacy when wearing a passive trunk exoskeleton. Furthermore, optimal fitting is essential if it comes to comparing results between participants. Due to the misalignment compensations and the adaptable hip frame of the exoskeleton [14], we believe that the fitting did not influence the results in this study. Another important point is the fact that we discussed factors to improve acceptability regarding the idea of having an exoskeleton that has been proven to support employees in their work by unloading their back and by that preventing from low-back pain. The SPEXOR exoskeleton was used as an example to discuss these issues here. Previous studies have shown that the SEPXOR exoskeleton reduces mechanical loading on the back [38], reduces metabolic cost during lifting [17] and improves functional performance in static forward bending tasks and lifting [16]. Still, the SPEXOR exoskeleton is not ready to be used in practice, but was used as an example to assess important factors that can affect successful implementation in the working environment.

## Conclusion

In this study, a mixed methods approach was used, performing quantitative measurements on self-efficacy, and conducting a focus group and an interview with employees suffering from low-back pain and managers from the same company. As self-efficacy is valuable for successful implementation of a device, we assessed the effect of the SPEXOR exoskeleton on self-efficacy using the modified spinal function sort (M-SFS). The findings demonstrate that the exoskeleton increases self-efficacy, especially for people feeling strongly restricted by their low-back pain. The focus group and interview confirm and complement these results, demonstrating the flexibility of the exoskeleton as an advantage to current static and inflexible external lifting devices and the perceived support during main tasks of employees. Managers see potential in using the exoskeleton as a preventive measure, whereas employees would only use it if suffering from low-back pain. To increase the acceptability of an exoskeleton at work sites, managers need to take into account factors that potentially affect the employees’ openness to use the exoskeleton. As described in this study, one factor might be being seen with the exoskeleton in a working environment, especially if this is dominated by a male culture. By wearing the exoskeleton, the user is seen to show vulnerability to colleagues, which could inhibit the use of the device. A contractual obligation was discussed to solve that problem. In that way, the exoskeleton could also be prescribed to people without low-back pain, who are less open to use it. If these potential challenges are considered, acceptability of the device to employees can be improved. Obviously, exoskeleton design may also play a role in dealing with this kind of barriers. By improving acceptability by following design requirements, acceptance of a passive trunk exoskeleton would be further improved, potentially contributing to reduced risk of low-back pain.

## Figures and Tables

**Fig. 5:**
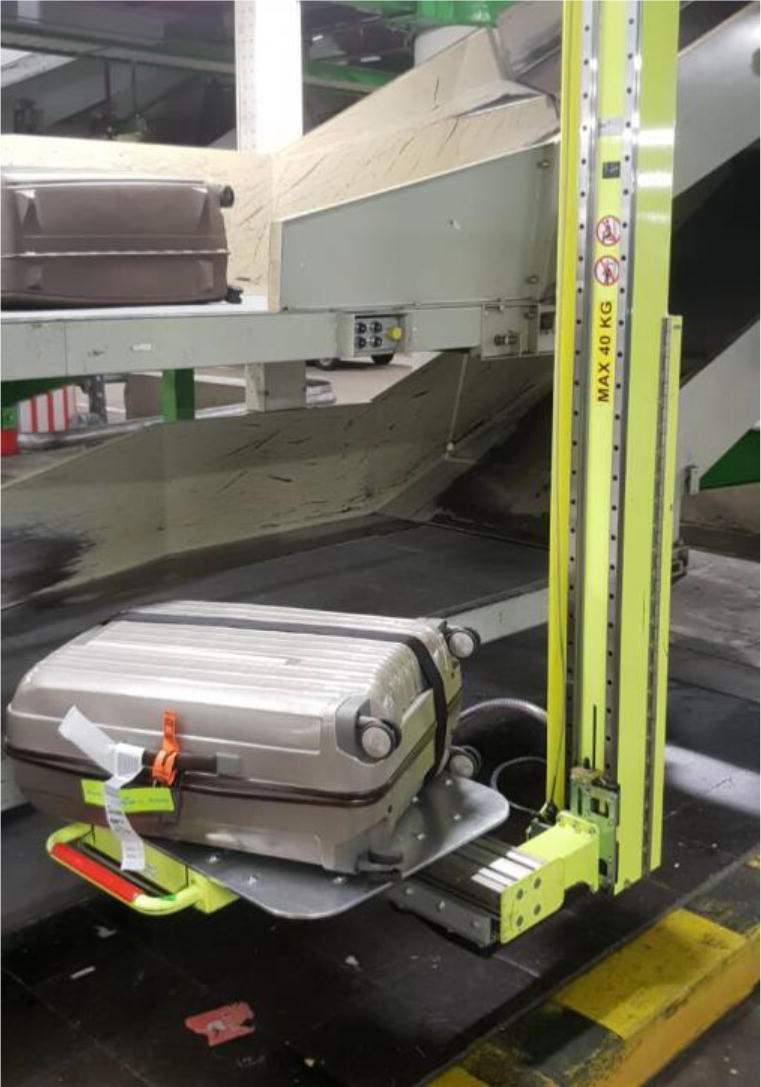
Adjustable lifting table

**Fig. 6:**
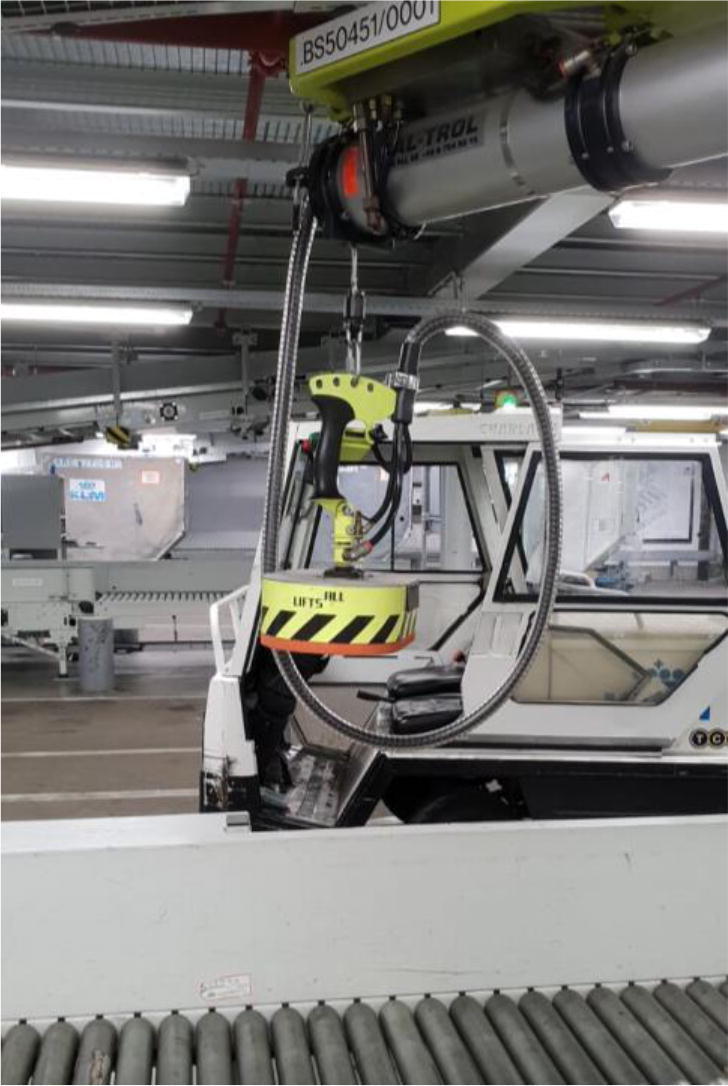
Vacuum lifting device. Not allowed to be used anymore

## Appendix

Two lifting devices are available at KLM. One is a lifting table, that can adjusted in height to pull the suitcases on and off (Fig. 5). The second one works with vacuum that sucks up the suitcase and by pressing a button releases the suitcase again (Fig. 6). This one is not allowed to be used anymore, since it led to various accidents.
